# Association between obesity and trastuzumab-related cardiac toxicity in elderly patients with breast cancer

**DOI:** 10.18632/oncotarget.17808

**Published:** 2017-05-11

**Authors:** Hai-Yan Wang, Bei-Bei Yin, Dan-Yan Jia, Ying-Long Hou

**Affiliations:** ^1^ Department of Echocardiography, Shandong Provincial Qianfoshan Hospital, Shandong University, Jinan, Shandong Province, China; ^2^ Department of Oncology, Shandong Provincial Qianfoshan Hospital, Shandong University, Jinan, Shandong Province, China; ^3^ Jinan Medical Emergency Center, Jinan, Shandong Province, China; ^4^ Department of Cardiology, Shandong Provincial Qianfoshan Hospital, Shandong University, Jinan, Shandong Province, China

**Keywords:** obesity, breast cancer, elderly patients, trastuzumab, cardiac toxicity

## Abstract

**Purpose:**

Trastuzumab can improve the prognosis for patients with breast cancer, but its related cardiac toxicity is concerning. This study aimed to identify the risk factors associated with trastuzumab-related cardiac toxicity in elderly patients with HER2-positive breast cancer.

**Patients and methods:**

A total of 133 elderly (≥ 65 years) patients who were diagnosed with breast cancer between June 1, 2007, and January 31, 2016, and received trastuzumab treatment were retrospectively reviewed. Cardiac events were defined as: (1) LVEF reduction of >10% from baseline echocardiography, (2) reduction of LVEF to <50%, and (3) signs and symptoms of heart failure as defined by the Common Terminology Criteria for Adverse Events (CTCAE) accompanied by a decrease in the LVEF. Univariate and multivariate regression analyses were used to determine the contribution of different clinical variables to trastuzumab-related cardiac events.

**Results:**

The median age of the cohort was 71.0 years (range, 65–81 years). The median follow-up period for measurement of left ventricular ejection fraction was 11.0 months (range, 2–71 months). Fifteen patients (11.2%) experienced cardiac events during the follow-up. Multivariate regression analysis revealed that obesity (odd ratio[OR], 4.706; 95% CI, 1.984-10.147; *P* = 0.002) was a statistically significant risk factor associated with cardiac events.

**Conclusion:**

Obesity is an independent risk factor for trastuzumab-related cardiac toxicity in elderly patients with breast cancer, receiving trastuzumab. Further studies are needed to establish the independent predictive value of obesity on cardiotoxicity in these patients.

## INTRODUCTION

Breast cancer is one of the most common malignant tumors and is estimated to account for almost 29% of all new cancer cases among women [1]. The incidence of breast cancer is directly proportional to aging, and approximately 40–50% of women diagnosed with breast cancer are older than 65 years [2, 3]. Breast cancer in elderly patients often presents with distinctive biological characteristics including absence of *p53* mutations, presence of hormone receptors, luminal molecular subtype, and low proliferative indices [4, 5]. Furthermore, elderly patients with breast cancer are more likely to present with more advanced tumors, and the risk for lymph node metastasis increases with age [6]. Elderly patients with breast cancer could be at a high risk of toxicity due to the decline in hematologic reserve, decrease in cognitive function, and high number of comorbidities [7]. In addition, treatment-related toxicity could be enhanced at an advanced age [8]. Due to the abovementioned reasons, the concerns of treatment-related toxicity in elderly patients with breast cancer are growing.

The gene for the human epidermal growth factor receptor2 (HER2) encodes a transmembrane ligand-activated tyrosine kinase receptor protein and its expression is noted in approximately 25–30% of breast cancer patients. Trastuzumab (Herceptin; Genentech, South San Francisco, CA), developed as a humanized monoclonal antibody and directed against the extracellular domain of the HER-2 receptor, has been confirmed to significantly improve overall survival and progression-free survival in patients with HER2-overexpressed breast cancer, including early, locally advanced and metastatic breast cancer [9, 10]. However, several studies have reported that trastuzumab, especially when combined with anthracyclines, is significantly associated with an increased risk of cardiotoxicity [11–13]. The risk-benefit ratio of trastuzumab in the elderly population has not yet been well defined. In fact, the geriatric population is usually under-represented in clinical trastuzumab trials [14–16].

Given the survival benefit of trastuzumab and the lack of known risk factors for cardiac toxicity in elderly breast cancer patients, the aim of this study was to assess the cardiac toxicity of trastuzumab in this population.

## RESULTS

A total of 133 patients diagnosed with breast cancer and aged ≥ 65 years were analyzed. Patient demographics and baseline characteristics are shown in Table 1. The median age of the entire cohort was 71.0 years (range, 65–81 years). The Eastern Cooperative Oncology Group performance status at the initiation of trastuzumab-based treatment was 0 in 79 patients (59.3%), 1 in 29 patients (21.8%), and 2 in 25 patients (18.8%), respectively. In addition, 41 patients (30.8%) had one comorbidity, 12 patients (9.0%) had two comorbidities, and 6 patients (4.5%) had ≥3 concomitant comorbidities. Among the 41 patients, 5 patients (3.8%) had a history of cardiac diseases. The median Charlson’s weighted index of comorbidities (WIC) in the whole cohort was 0 (range, 0–8). A total of 37 patients (27.8%) were obese (body mass index [BMI] ≥ 30 kg/m^2^), 31 patients (23.3%) had a BMI of 25.0–29.9 30 kg/m^2^, and 65 patients (48.9%) had a BMI < 25 kg/m^2^. In the whole cohort, the mean cumulative dose of anthracyclines was 116 mg/m^2^ (ranging 0-540). A total of 36 patients were already being treated for arterial hypertension throughout the treatment period of trastuzumab: beta-adrenergic blocker (14 patients), ACEI/ARBs (8 patients), both (3 patients) and other medications (11 patients). The radiotherapy was delivered to the chest wall in 18 patients (13.5%) and one patient received mantle field irradiation for Hodgkin’s lymphoma. The median radiation dose of chest wall was 50 Gy (ranging from 40 to 60Gy).

**Table 1:** Patient characteristics

Characteristics	No. of patients (%)
Age (years)	
Median (range)	71.0 (65.0-81.0)
ECOG performance status	
0-1	108 (81.2)
≥ 2	25 (18.8)
BMI (kg/m^2^)	
< 30	96 (72.2)
≥ 30	37 (27.8)
WIC	
≥ 1	52 (39.1)
< 1	81 (60.9)
Baseline LVEF	
Median (range)	61% (47.0–69.0)
TNM stage	
I-II	39 (29.3)
III	61 (45.9)
IV	33 (24.8)
Anthracycline exposure	
Previous	49 (36.8)
Concomitant	22 (16.5)
None	62 (46.6)
Anthracycline cumulative dose (mg/m2)	116 (0-540)
Previous radiotherapy (thoracic wall)	
Yes	19 (14.3)
No	124 (93.2)

*Abbreviations*: ECOG, Eastern Cooperative Oncology Group; WIC, Charlson’s weighted index of comorbidities; LVEF, left ventricular ejection fraction; CT, chemotherapy

**Figure 1:**
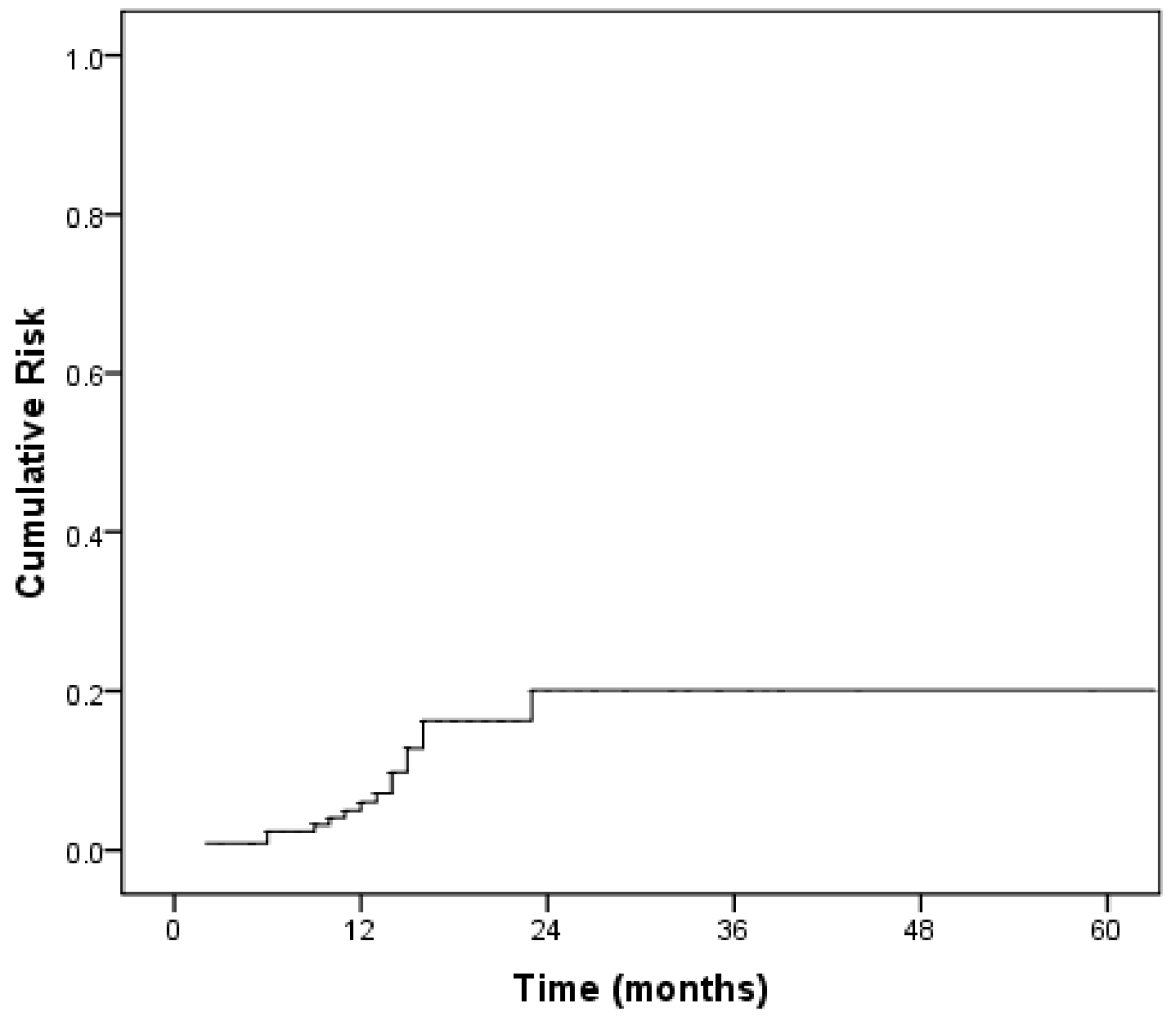
The cumulative risk of cardiac toxicity in the study cohort

The left ventricular ejection fraction (LVEF) value was determined at baseline, and the median follow-up of LVEF value was 11.0 months (range, 2–71 months). The median LVEF value at baseline was 61% (range, 47.0–69.0). Fifteen patients (11.2%) experienced a cardiac event during the follow-up period. Of them, 8 (8/15, 53.3%) and 6 (6/15, 40%) patients developed grade 1 and grade 2 LVEF dysfunction, respectively. Only one patient (1/15, 6.6%) presented with symptomatic chronic heart failure and received trastuzumab in combination with liposomal doxorubicin plus paclitaxel for metastatic disease. None of the patients experienced cardiac death during the follow-up. The median time to onset of a cardiac event was 10.0 months (range, 2–26 months), with a median time to recovery of 3 months. Figure 1 shows the cumulative risk of cardiac events in this cohort.

There were 71 patients who received the combination of anthracyclines and trastuzumab (prior to trastuzumab: 49 patients; concomitant trastuzumab: 22 patients). In the 71 patients, there were a total of 11 patients experienced a cardiac event during the follow-up period. In the 10 patients, 7 and 3 patients developed grade 1 and grade 2 LVEF dysfunction, respectively. One patient experienced symptomatic chronic heart failure as mentioned above. In the 71 patients, the dose of anthracyclines were not significantly associated with the cardiac toxicity (high dose *vs.* low dose, logistic regression analyses *P*=0.939). The high dose of anthracyclines means doxorubicin>240 mg/m2 or epirubicin > 500 mg/m2. There were 48 patients receiving low dose of anthracyclines with 8 patients experiencing cardiac events. The rest 23 patients received high dose of anthracyclines with 4 patients experiencing cardiac events.

**Figure 2:**
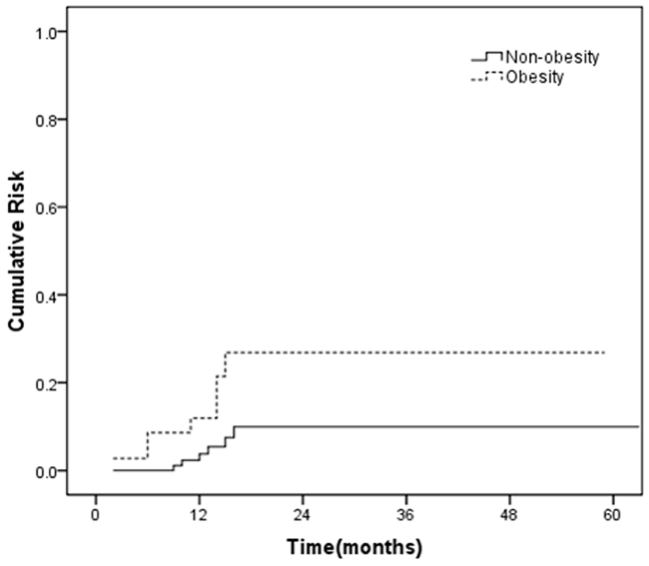
The cumulative risk of cardiac toxicity for patients with *vs*. without obesity (*P* = 0.040)

Univariate analysis revealed that the cardiac events were significantly associated with obesity (P = 0.006) and anthracycline exposure (P = 0.039) (Table 2). A history of radiotherapy (P = 0.093) and WIC (P = 0.086) were of borderline significance in the univariate analysis. To identify independent predictive risk factors, the factors found to be significant in univariate analysis (P < 0.10) were included in the multivariate analysis. Multivariate regression analysis revealed that obesity(odd ratio[OR], 4.706; 95% CI, 1.984-10.147; *P* = 0.002) was significantly associated with cardiac toxicity after adjusting for confounding variables (Table 3). As is shown in Figure 2, the cumulative risk curve of cardiotoxicity by obesity showed that the obese patients had a significant high risk of cardiotoxicity compared with those patients without obesity (*P*= 0.040).

**Table 2:** Univariate regression analyses of risk factors for cardiac events

Risk factors	Patients,(%)	Cardiac events	OR	95% CI	*P*
Age					
> 71 years	65 (48.9)	9 (13.8)	0.702	0.338-1.799	0.364
≤ 71 years	68 (51.1)	6 (8.8)			
Performance status					
≤ 1	108 (81.2)	11 (10.2)	1.112	0.752-1.328	0.628
> 1	25 (18.8)	4 (16.0)			
WIC					
≥ 1	52 (39.1)	9 (17.3)	0.382	0.127-1.147	0.086
< 1	81 (60.9)	6 (7.4)			
Hypertension					
Yes	36 (27.1)	5 (13.9)	1.403	0.445-4.428	0.563
No	97 (72.9)	10 (10.3)			
History of cardiac diseases					
Yes	5 (3.8)	1 (20.0)	0.491	0.151-2.710	0.538
No	128 (96.2)	14 (10.9)			
Obesity					
Yes	37 (27.8)	9 (24.3)	3.821	1.578-8.728	0.006
No	96 (72.2)	6 (6.2)			
TNM stage					
I-III	100 (75.2)	9 (9.0)	2.247	0.734-6.675	0.156
IV	33 (24.8)	6 (18.1)			
Anthracycline exposure					
Yes	71 (53.4)	12 (16.9)	0.250	0.067-0.932	0.039
No	62 (46.6)	3 (4.8)			
Duration of trastuzumab					
≥ 40 weeks	68 (51.1)	8 (11.8)	0.905	0.308-2.657	0.856
< 40 weeks	65 (48.9)	7 (10.8)			
Previous radiotherapy					
No	57 (42.9)	4 (7.0)	0.431	0.149-1.469	0.093
Yes	76 (57.1)	11 (14.8)			
Hormone therapy					
Yes	19 (14.3)	4 (21.1)	0.370	0.154-1.322	0.126
No	124 (93.2)	11 (8.9)			
Receiving surgery					
Yes	61 (45.9)	6 (9.8)	1.266	0.424-3.782	0.673
No	72 (54.1)	9 (12.5)			

*Abbreviations*: OR, odds ratio; 95% CI, 95% confidence interval; WIC, Charlson’s weighted index of comorbidities.

**Table 3:** Multivariate regression analyses of risk factors for cardiac events

Risk factors	OR	95% CI for OR	*P* value
WIC	0.891	0.222-2.715	0.195
Obesity	4.706	1.984-10.147	0.002
Anthracycline exposure	0.268	0.075-1.116	0.065
Previous radiotherapy	1.095	0.189-5.363	0.919

*Abbreviations*: OR, odds ratio; 95% CI, 95% confidence interval; WIC, Charlson’s weighted index of comorbidities.

## DISCUSSION

Trastuzumab is the current standard of treatment in HER-2–positive breast cancer patients [17]. However, reports on the cardiac toxicity of trastuzumab-based treatments in elderly patients with breast cancer are limited. Furthermore, although approximately 40% of patients with newly diagnosed breast cancer are older than 65 years [3], the geriatric population is usually under-represented in clinical trials due to their severe comorbid conditions, poor life expectancy, and treatment intension. Therefore, this study evaluated the trastuzumab-related cardiac toxicity in such patients.

Trastuzumab has been shown to significantly improve the overall survival and response rate of breast cancer patients with HER-2 over-expression, regardless of TNM stages [9, 10]. A meta analysis which included eight randomised controlled trials involving 11,991 patients, assessed the efficacy of trastuzumab-containing regimens in patients with early and locally advanced breast cancer. The result showed that the trastuzumab-containing regimens could significantly improve the prognosis in these patients [10]. The similar results were achieved in the meta analysis which aimed to evaluate the survival benefit of trastuzumab in patients with metastatic breast cancer [9]. Slamon et al. prospectively evaluated the efficacy and safety of addition of trastuzumab to chemotherapy for patients with HER2-overexpressing metastatic breast cancer; their results showed that in comparison with chemotherapy alone, the addition of trastuzumab to chemotherapy significantly improved the prognosis (median survival, 20.3 months *vs.* 25.1 months; *P* = 0.046) and the objective response rates (32% *vs.* 50 %, *P* < 0.001) [11]. However, unexpected adverse events were observed during the pivotal trials of trastuzumab. Numerous studies have provided evidence of trastuzumab-related cardiotoxicity [12–14, 18–20]. The mechanism that trastuzumab, as an inhibitory ErbB2 antibody, could increase the risk of development of cardiotoxicity is not completely clear so far. One of mechanistic theories of trastuzumab-related cardiotoxicity was the ‘dual-hit’ mechanism [21]. On one hand, trastuzumab could directly inhibit HER2’s ability to dimerize and signal cell survival through MAPK/ERK 1/2, phosphoinositide 3 kinase (PI3K)/AKT and FAK-dependent pathways. Thus, the cardiomyocytes could not activate the cell survival pathways to cope with the excess reactive oxygen species (ROS), which could stimulate cardiomyocyte apoptosis to promote the development of cardiac dysfunction [21, 22]. On the other hand, trastuzumab could upregulate angiotensin II (ANG II), a potent inhibitor of neuregulin (NRG). The ANG II not only could downregulate and prevent NRG-1 from binding to HER2/4 to initiate cell survival pathways, but also could promote ROS production by the activation of NADPH oxidase [21, 23]. Furthermore, the addition of chemotherapy, especially anthracycline, could aggravate trastuzumab-related cardiotoxicity. The synergy between trastuzumab and anthracycline could potentiate the increase of oxidative stress which could contribute to cardiac dysfunction, by up-regulating the formation of ROS and reducing antioxidants [21]. A large review including data from 1219 patients with breast cancer revealed the increased risk of cardiac toxicity in patients receiving trastuzumab concomitantly combined with an anthracycline derivative and cyclophosphamide [12]. Slamon et al. enrolled 3222 patients with HER2-positive early breast cancer and reported that the combination of trastuzumab and chemotherapy led to a high incidence of cardiac events. The incidence of subclinical decrease in LVEF was 18.6% in patients administered doxorubicin, paclitaxel, cyclophosphamide, and trastuzumab; 9.4% in patients administered docetaxel, carboplatin, and trastuzumab; and 11.2% in patients administered doxorubicin, cyclophosphamide, and paclitaxel [24].

The issue of cardiac safety of drugs has gained more attention recently. In the first prospective pivotal study, Slamon et al reported that up to 27% of patients treated with trastuzumab with anthracycline and cyclophosphamide had either symptomatic heart failure or asymptomatic cardiac dysfunction [11]. The meta analysis, which involving 11,991 patients with early and locally advanced breast cancer, reported that patients treated with trastuzumab was significantly associated with higher risk of LVEF decline and severe cardiac event, compared with the control group [10]. In metastatic breast cancer patients, the meta analysis also revealed that those treated with trastuzumab had significant increasing risk of LVEF decline and severe cardiac event in comparison with the control group [9]. Previous study has revealed that the increasing risk of cardiac toxicity in patients received with trastuzumab was independent of TNM stage [25]. Du et al reported the rates of cardiac toxicity among a heterogeneous population of breast patients with stage I-IV (n = 880). Compared with patients not receiving chemotherapy, those who received trastuzumab in combination with an anthracycline (HR, 2.37; 95% CI, 1.76 - 3.19), or trastuzumab alone (HR, 1.97; 95% CI, 1.46-2.67) both had a significantly higher risk of symptomatic congestive heart failure [25]. The elderly patients have been consistently underrepresented in clinical trials and, therefore, the risk of trastuzumab-related cardiac toxicities has not clearly clarified in this patient population so far. The increasing age has been revealed as the only significant risk factor associated with cardiac dysfunction in the first prospective pivotal study [11]. The subsequent analysis of the prospective Herceptin Adjuvant (HERA) trial that included patients with early stage invasive breast cancer receiving adjuvant trastuzumab reported 10 cardiac events in the subgroup of 267 patients aged ≥60 years [20]. In a study with large cohort of older breast cancer, Chavez-MacGregor et al retrospectively studied 2,203 elderly patients with breast cancer who received trastuzumab and revealed that the rate of congestive heart failure was 29.4%. The old age of their cohort was a significantly high risk factor of congestive heart failure [26]. Serrano et al retrospectively reviewed 45 breast cancer patients (25 patients with stage I-III and 20 patients with stage IV) who were older than 70 years and treated with trastuzumab to assess the cardiac safety of trastuzumab in elderly breast cancer patients. During the following-up, twelve patients (26.7%) experienced a cardiac event, with 4 patients being symptomatic. The authors concluded that elderly breast cancer patients (> 70 years) with a history of cardiac disease and/or diabetes had an increased risk of cardiac toxicity when treated with trastuzumab [27]. In the clinical practice of breast cancer, McArthur et al analyzed 102 patients with breast cancer who were treated with trastuzumab, and the incidence of trastuzumab discontinuation was 21.6% due to cardiac toxicity [28]. In our study, we focused on evaluating the trastuzumab-related cardiac events as a measure of its safety in elderly patients with breast cancer. The overall incidence of cardiac events in this study was 11.3%. The cardiac toxicity, especially subclinical cardiomyopathies, has been recognized as an important side effect of trastuzumab therapy. The European Society of Cardiology Committee recommended that the typically thorough cardiac monitoring should be performed every 3 months during and once after completion of trastuzumab therapy [29]. They also advised that high-risk survivors should undergo measurement of troponin with every cycle [29].

To the best of our knowledge, this study was the first to address the value of obesity in trastuzumab-related cardiac toxicity in an elderly cohort of breast cancer. The association between obesity (BMI ≥ 30 kg/m^2^) and high risk of heart failure has been well established: A large, epidemiologic study which analyzed a community-based sample of 5,881 individuals revealed that an increased BMI was significantly associated with an increased risk of heart failure [30]. In addition, previous studies demonstrated that obesity was an important prognostic factor that affected both overall survival and disease-free survival in patients with breast cancer [31]. In our study, obesity had an important role in trastuzumab-related cardiac events. The mechanisms by which obesity could negatively influence cardiotoxicity are affected by numerous confounding factors. First, obesity may increase the expression of pro-inflammatory adipokines and downregulate the anti-inflammatory adipokines, which could result in an adipokine imbalance and maintain a chronic inflammatory state to promote the development of cardiovascular diseases [32]. Second, patients with obesity are more sensitive to the cardiotoxic effects of anthracyclines; an animal model of rats with a high-lipid diet showed similar results [33]. Finally, obesity is significantly associated with activation of neurohormones, increased oxidative stress, increased hemodynamic load, and remodeling of the left ventricle [34]. Further studies should be performed to elucidate the role of obesity in trastuzumab-related cardiac toxicity. Other cardiac event-related factors, including WIC, history of cardiac diseases, anthracycline exposure, and previous history of radiotherapy were not significantly associated with trastuzumab-related cardiac events in this study.

Despite our important findings, this study has several limitations that need to be addressed. First, the study was retrospective in nature and included only one institution. Second, the follow-up duration was short and the sample size was small. Thus, a potential lack of power to detect small differences as a result of population size and the retrospective nature might have influenced the accuracy of cardiotoxicity rates. On the other hand, the decrease in LVEF alone, as a parameter of cardiac toxicity, is considered relatively inadequate for surveillance of cardiac toxicity in patients with breast cancer. At last, the main limitation of 2D echocardiography included its relatively moderate reproducibility, which was inevitably derived from the different equipment, intra- and inter-observer variability. But it should be noted that the 2D echocardiography has been recommended as one of diagnostic tools for the detection of cardiotoxicity by the European Society of Cardiology (ESC) committee, due to the wide availability and the ability to assess haemodynamics and other cardiac structures [29].

In conclusion, obesity was an independent risk factor for cardiac toxicity in elderly patients with breast cancer, receiving trastuzumab. A careful assessment of cardiovascular risk factors is important to improve risk stratification for cardiotoxicity in elderly patients with breast cancer. Further studies are needed to establish the independent predictive value of obesity on cardiotoxicity in these patients.

## PATIENTS AND METHODS

This was a retrospective study of 133 elderly patients diagnosed with breast cancer and treated in a Shandong Provincial Qianfoshan Hospital between June 1, 2007, and January 31, 2016. The inclusion criteria were as follows: (1) histologically confirmed breast cancer, (2) overexpression of HER2 indicated by immunohistochemistry or gene amplification by fluorescence in situ hybridization or chromogenic in situ hybridization, according to the American Society of Clinical Oncology/College of American Pathologists guidelines, (3) age ≥ 65 years, and (4) receipt of at least one dose of trastuzumab as a part of treatment. The clinicopathologic characteristics and treatment-related factors were extracted retrospectively from the medical records for each patient. Written informed consent was obtained from all patients, and approval for the study was obtained from the independent Institute Research Ethics Committee at the local hospital.

Cardiac events were defined as the presence of one of the following [35]: (1) LVEF reduction of >10% from baseline echocardiography, (2) reduction of LVEF to <50%, and (3) signs and symptoms of heart failure as defined by the Common Terminology Criteria for Adverse Events (CTCAE) accompanied by a decrease in the LVEF. Trastuzumab was discontinued in patients who experienced cardiac events. In other cases, the decision of continuing trastuzumab was considered on a case-by-case basis, following close cardiac monitoring. The WIC was applied to provide a descriptive analysis of the cohort’s comorbidity burden. The WIC is a standardized and validated tool that allows systematic ascertainment of comorbidities and their effect on patient’s mortality.

All patients routinely received a comprehensive baseline physical examination and echocardiography as pre-chemotherapy evaluation. The cardiac examination and echocardiography were repeated every 3 months during trastuzumab treatment. Trastuzumab was administered weekly (first cycle of 4 mg/kg followed by 2 mg/kg) or every 3 weeks (first cycle of 8 mg/kg followed by 6 mg/kg).

### Statistical analysis

Continuous variables were reported by mean and standard deviation. Univariate and multivariate regression analyses were used to determine the contribution of different clinical variables to the trastuzumab-related cardiac events. The overall cumulative risk of cardiac events was estimated by the Kaplan-Meier method. The last follow-up evaluation was performed in October 2016. All statistical analyses were performed using PASW Statistics 21 (SPSS Inc., Chicago, IL, USA). A two-sided *P* value < 0.05 was considered statistically significant.

## Abbreviations

HER2: human epidermal growth factor receptor2
WIC: Charlson’s weighted index of comorbidities
BMI: body mass index
LVEF: left ventricular ejection fraction
CTCAE: Common Terminology Criteria for Adverse Events

## References

[1] Siegel RL, Miller KD, Jemal A (2015). Cancer Statistics, 2015. CA Cancer J Clin.

[2] Marshall SF, Clarke CA, Deapen D, Henderson K, Largent J, Neuhausen SL, Reynolds P, Ursin G, Horn-Ross PL, Stram DO, Templeman C, Bernstein L (2010). Recent breast cancer incidence trends according to hormone therapy use: the California Teachers Study cohort. Breast Cancer Res.

[3] Wildiers H, Kunkler I, Biganzoli L, Fracheboud J, Vlastos G, Bernard-Marty C, Hurria A, Extermann M, Girre V, Brain E, Audisio RA, Bartelink H, Barton M (2007). Management of breast cancer in elderly individuals: recommendations of the international society of geriatric oncology. Lancet Oncol.

[4] Ebner F, van Ewijk R, Wöckel A, Hancke K, Schwentner L, Fink V, Kreienberg R, Janni W, Blettner M (2015). Tumor biology in older breast cancer patients—what is the impact on survival stratified for guideline adherence? A retrospective multi-centre cohort study of 5378 patients. Breast.

[5] Carey LA, Perou CM, Livasy CA, Dressler LG, Cowan D, Conway K, Karaca G, Troester MA, Tse CK, Edmiston S, Deming SL, Geradts J, Cheang MC (2006). Race, breast cancer subtypes, and survival in the Carolina Breast Cancer Study. JAMA.

[6] Gennari R, Curigliano G, Rotmensz N, Robertson C, Colleoni M, Zurrida S, Nolè F, de Braud F, Orlando L, Leonardi MC, Galimberti V, Intra M, Veronesi P (2004). Breast carcinoma in elderly women-features of disease presentation, choice of local and systemic treatments compared with younger postmenopausal patients. Cancer.

[7] Wildiers H (2008). Challenges in treating older cancer patients: breast cancer. Ann Oncol.

[8] Muss HB, Berry DA, Cirrincione CT, Theodoulou M, Mauer AM, Kornblith AB, Partridge AH, Dressler LG, Cohen HJ, Becker HP, Kartcheske PA, Wheeler JD, Perez EA (2009). Adjuvant chemotherapy in older women with early–stage breast cancer. N Engl J Med.

[9] Balduzzi S, Mantarro S, Guarneri V, Tagliabue L, Pistotti V, Moja L, D’Amico R (2014). Trastuzumab-containing regimens for metastatic breast cancer. Cochrane Database Syst Rev.

[10] Moja L, Tagliabue L, Balduzzi S, Parmelli E, Pistotti V, Guarneri V, D’Amico R (2012). Trastuzumab containing regimens for early breast cancer. Cochrane Database Syst Rev.

[11] Slamon DJ, Leyland-Jones B, Shak S, Fuchs H, Paton V, Bajamonde A, Fleming T, Eiermann W, Wolter J, Pegram M, Baselga J, Norton L (2001). Use of chemotherapy plus a monoclonal antibody against HER2 formetastatic breast cancer that overexpresses HER2. N Engl J Med.

[12] Seidman A, Hudis C, Pierri MK, Shak S, Paton V, Ashby M, Murphy M, Stewart SJ, Keefe D (2002). Cardiac dysfunction in the trastuzumab clinical trials experience. J Clin Oncol.

[13] Costa RB, Kurra G, Greenberg L, Geyer CE (2010). Efficacy and cardiac safety of adjuvant trastuzumab-based chemotherapy regimens for HER2-positive early breast cancer. Ann Oncol.

[14] Gianni L, Dafni U, Gelber RD, Azambuja E, Muehlbauer S, Goldhirsch A, Untch M, Smith I, Baselga J, Jackisch C, Cameron D, Mano M, Pedrini JL (2011). Treatment with trastuzumab for 1 year after adjuvant chemotherapy in patients with HER2-positive early breast cancer: a 4-year follow-up of a randomized controlled trial. Lancet Oncol.

[15] Romond EH, Perez EA, Bryant J, Suman VJ, Geyer CE, Davidson NE, Tan-Chiu E, Martino S, Paik S, Kaufman PA, Swain SM, Pisansky TM, Fehrenbacher L (2005). Trastuzumab plus adjuvant chemotherapy for operable HER2-positive breast cancer. N Engl J Med.

[16] Spielmann M, Roché H, Delozier T, Canon JL, Romieu G, Bourgeois H, Extra JM, Serin D, Kerbrat P, Machiels JP, Lortholary A, Orfeuvre H, Campone M (2009). Trastuzumab for patients with axillarynode- positive breast cancer: results of the FNCLCC-PACS 04 trial. J Clin Oncol.

[17] Carlson RW, Allred DC, Anderson BO, Burstein HJ, Carter WB, Edge SB, Erban JK, Farrar WB, Goldstein LJ, Gradishar WJ, Hayes DF, Hudis CA, Jahanzeb M (2009). Breast cancer. Clinical practice guidelines in oncology. J Natl Compr Canc Netw.

[18] Moslehi JJ (2016). Cardiovascular toxic effects of targeted cancer cherapies. N Engl J Med.

[19] Naumann D, Rusius V, Margiotta C, Nevill A, Carmichael A, Rea D, Sintler M (2013). Factors Predicting Trastuzumab-related Cardiotoxicity in a Real-world Population of Women with HER2+ Breast Cancer. Anticancer Res.

[20] Suter TM, Procter M, van Veldhuisen DJ, Muscholl M, Bergh J, Carlomagno C, Perren T, Passalacqua R, Bighin C, Klijn JG, Ageev FT, Hitre E, Groetz J (2007). Trastuzumab-associated cardiac adverse effects in the herceptin adjuvant trial. J Clin Oncol.

[21] Zeglinski M, Ludke A, Jassal DS, Singal PK (2011). Trastuzumab-induced cardiac dysfunction: A ‘dual-hit’. Exp Clin Cardiol.

[22] Gordon LI, Burke MA, Singh AT, Prachand S, Lieberman ED, Sun L, Naik TJ, Prasad SV, Ardehali H (2009). Blockade of the erbB2 receptor induced cardiomyocyte death through mitochondrial and reactive oxygen species-dependent pathways. J Biol Chem.

[23] Lemmens K, Doggen K, De Keulenaer GW (2007). Role of neuregulin-1/ErbB signaling in cardiovascular physiology and disease: implications for therapy of heart failure. Circulation.

[24] Slamon D, Eiermann W, Robert N, Pienkowski T, Martin M, Press M, Mackey J, Glaspy J, Chan A, Pawlicki M, Pinter T, Valero V, Liu MC (2011). Adjuvant trastuzumab in HER2-positive breast cancer. N Engl J Med.

[25] Du XL, Xia R, Burau K, Liu CC (2011). Cardiac risk associated with the receipt of anthracycline and trastuzumab in a large nationwide cohort of older women with breast cancer, 1998-2005. Med Oncol.

[26] Chavez-MacGregor M, Zhang N, Buchholz TA, Zhang Y, Niu J, Elting L, Smith BD, Hortobagyi GN, Giordano SH (2013). Trastuzumab-related cardiotoxicity among older patients with breast cancer. J Clin Oncol.

[27] Serrano C, Cortés J, De Mattos-Arruda L, Bellet M, Gómez P, Saura C, Pérez J, Vidal M, Muñoz-Couselo E, Carreras MJ, Sánchez-Ollé G, Tabernero J, Baselga J, Di Cosimo S (2012). Trastuzumab-related cardiotoxicity in the elderly: a role for cardiovascular risk factors. Ann Oncol.

[28] McArthur HL, Chia S (2007). Cardiotoxicity of trastuzumab in clinical practice. N Engl J Med.

[29] Zamorano JL, Lancellotti P, Rodriguez Muñoz D, Aboyans V, Asteggiano R, Galderisi M, Habib G, Lenihan DJ, Lip GY, Lyon AR, Lopez Fernandez T, Mohty D (2017). 2016 ESC Position Paper on cancer treatments and cardiovascular toxicity developed under the auspices of the ESC Committee for Practice Guidelines: The Task Force for cancer treatments and cardiovascular toxicity of the European Society of Cardiology (ESC). Eur J Heart Fail.

[30] Kenchaiah S, Evans JC, Levy D, Wilson PW, Benjamin EJ, Larson MG, Kannel WB, Vasan RS (2002). Obesity and the risk of heart failure. N Engl J Med.

[31] Ladoire S, Dalban C, Roche´ H, Spielmann M, Fumoleau P, Levy C, Martin AL, Ecarnot F, Bonnetain F, Ghiringhelli F (2014). Effect of obesity on disease-free and overall survival in node positive breast cancer patients in a large French population: A pooled analysis of two randomised trials. Eur J Cancer.

[32] Nakamura K, Fuster JJ, Walsh K (2014). Adipokines: A link between obesity and cardiovascular disease. J Cardiol.

[33] Mitra MS, Donthamsetty S, White B, Mehendale HM (2008). High fat diet-fed obese rats are highly sensitive to doxorubicin-induced cardiotoxicity. Toxicol Appl Pharmacol.

[34] Engeli S, Sharma AM (2001). The renin-angiotensin system and natriuretic peptides in obesity-associated hypertension. J Mol Med.

[35] Hunt SA, American College of Cardiology; American Heart Association Task Force on Practice Guidelines (Writing Committee to Update the 2001 Guidelines for the Evaluation and Management of Heart Failure) (2005). ACC/AHA 2005 guideline update for the diagnosis and management of chronic heart failure in the adult: a report of the American College of Cardiology/American Heart Association Task Force on Practice Guidelines (Writing Committee to Update the 2001 Guidelines for the Evaluation and Management of Heart Failure). J Am Coll Cardiol.

